# Sanger sequencing as a first-line approach for molecular diagnosis of Andersen-Tawil syndrome

**DOI:** 10.12688/f1000research.11610.1

**Published:** 2017-06-28

**Authors:** Armando Totomoch-Serra, Manlio F. Marquez, David E. Cervantes-Barragán

**Affiliations:** 1 Department of Genetics and Molecular Biology, Centro de Investigación y de Estudios Avanzados del Instituto Politécnico Nacional, Ciudad de México, Mexico; 2 Department of Electrophysiology, Instituto Nacional de Cardiología “Ignacio Chávez”, Ciudad de México, Mexico; 3 Department of Genetics, Hospital Central del Sur de Alta Especialidad PEMEX, Ciudad de México, Mexico

**Keywords:** Sanger sequencing, Andersen-Tawil, KCNJ2, genetic testing, Next Generation Sequencing, clinical diagnosis, mutation

## Abstract

In 1977, Frederick Sanger developed a new method for DNA sequencing based on the chain termination method, now known as the Sanger sequencing method (SSM).  Recently, massive parallel sequencing, better known as next-generation sequencing (NGS),  is replacing the SSM for detecting mutations in cardiovascular diseases with a genetic background. The present opinion article wants to remark that “targeted” SSM is still effective as a first-line approach for the molecular diagnosis of some specific conditions, as is the case for Andersen-Tawil syndrome (ATS). ATS is described as a rare multisystemic autosomal dominant channelopathy syndrome caused mainly by a heterozygous mutation in the *KCNJ2 *gene *. *KCJN2 has particular characteristics that make it attractive for “directed” SSM. *KCNJ2* has a sequence of 17,510 base pairs (bp), and a short coding region with two exons (exon 1=166 bp and exon 2=5220 bp), half of the mutations are located in the C-terminal cytosolic domain, a mutational hotspot has been described in residue Arg218, and this gene explains the phenotype in 60% of ATS cases that fulfill all the clinical criteria of the disease. In order to increase the diagnosis of ATS we urge cardiologists to search for facial and muscular abnormalities in subjects with frequent ventricular arrhythmias (especially bigeminy) and prominent U waves on the electrocardiogram.

## Introduction

In 1977, Frederick Sanger developed a new method for DNA sequencing based on the chain termination method, where nucleotides in a single-stranded DNA molecules are determined by complementary synthesis of polynucleotide chains, based on the selective incorporation of chain-terminating dideoxynucleotides driven by the DNA polymerase enzyme ^1^. For this method, Sanger was awarded in 1980 with a second Nobel Prize in Chemistry, and nowadays this method is still known as the Sanger method of DNA sequencing, becoming a standard method in clinical genetics. The present opinion article wants to remark that, targeted SSM is still effective in specific clinical scenarios at a lower cost as a diagnostic method compared to new technologies for sequencing, one example is the detection of Andersen-Tawil syndrome (ATS).

## Next-generation sequencing: available for everyone?

Next-generation sequencing (NGS) technology, also known as massive parallel, high throughput or deep sequencing, is gradually replacing the traditional SSM as the first choice method for screening mutations in genetic cardiovascular diseases ^2^.

The genetic heterogeneity in long QT ^3^ and Brugada syndromes ^4^ has made this new genetic testing approach mandatory. The advantages of NGS versus the SSM in cases of genetic heterogeneity are undeniable, but NGS is still expensive and unaffordable for developing countries. The SSM remains the gold standard for sequencing short fragments of DNA (<1000 bases), previously amplified by PCR.

## Andersen-Tawil Syndrome: a rare disease

ATS, also named Long QT syndrome type 7, is described in the Online Mendelian Inheritance in Man database ( OMIM) as a multisystem autosomal dominant channelopathy syndrome caused by a heterozygous mutation in the *KCNJ2* gene (OMIM Entry - *600681) on chromosome 17q24.3. Periodic paralysis, ventricular arrhythmia, and distinctive dysmorphic features characterize it. Until 2015, the only gene thought to be affected was the potassium voltage-gated channel subfamily J member 2 ^5^ (the *KCNJ2* gene), which encodes the alpha subunit protein of the Kir2.1 channel composed of tetramers ^6^. Mutations in this gene have been reported in 60% of clinically suspected cases (which are classified as ATS type 1) ^7^. Less than 200 cases with the *KCNJ2* gene affected have been described worldwide since the discovery of the first mutations in 2001 ^8, 9^. In 2014, a novel variant (c.472A>G; p.Thr158Ala) in a second gene, *KCNJ5*, was associated with ATS in one Japanese patient taken from a cohort of 21 patients that had previously been screened negative for mutations in *KCNJ2*. The *KCNJ5* gene protein (potassium channel Kir 3.4 protein) has an interaction with the *KCNJ2* protein that leads to a dominant negative effect in the channel formed, related to the ATS phenotype ^10^. No additional cases of *KCNJ5* mutations in independent series of ATS patients have been reported; the frequency of *KCNJ5* mutations in ATS has to be determined in the future. With the widespread use of NGS, it is possible that in the next years we could discover new genes that explain part of the genetic heterogeneity observed in ATS, clarifying some of the 40% of clinically suspected negative cases that do not have a mutation in *KCNJ2* (nowadays classified as ATS type 2) ^11^.

## A special gene: *KCNJ2*

The *KCNJ2* gene has particular structural characteristics that makes it attractive for direct SSM, such as a relatively short sequence of 17,510 base pairs (bp), and a coding region with near to 5,000 bp with two exons (exon 1=166 bp and exon 2=5220 bp). Also, half of the mutations are located in the C-terminal cytosolic domain, and have a mutational hotspot in the residue Arg218; as we have addressed before, this gene explains the phenotype in 60% of ATS cases, fulfilling the clinical criteria.

## ATS in mestizo populations: the first description of ATS in the Mexican population

Fifteen years have passed since the first family with ATS in a Mexican population was reported by Canun *et al*. ^12^ Recently, a second proband was diagnosed in a different Mexican family, finding the mutation p.Arg218Trp in *KCNJ2* ^13^.

## Multidisciplinary approach: a productive collaboration

A multidisciplinary approach is extremely useful to study suspicious cases of hereditary sudden death syndrome. For ATS, the team must include a cardiologist, a neurologist and a clinical geneticist. It is very important that each of these physicians had expertise in the evaluation of subjects with sudden cardiac death syndrome. After a common agreement on suspicion of ATS, the whole coding region and intron boundaries of the non-coding region in *KCNJ2* could be sequenced with the SSM ^14^.

## Clinical ATS data that needs to be considered

Phenotypically, Canun *et al* ^11^ suggested that recognition of facial and limb dysmorphism (broad forehead, bushy eyebrows, small eyes, bulbous nose, malar and mandibular hypoplasia, crowded teeth, clinodactyly in the 5 ^th^ finger and cutaneous syndactyly in 2–3 toes) associated with ATS could help establish a correct ATS diagnosis. We believe that it is important that all cardiologists dealing with subjects with ventricular arrhythmias, specifically frequent ventricular premature beats in bigeminy, are aware of such distinctive phenotypic characteristics and also search for muscular disorders (weakness in limbs or periodic paralysis).

## Sanger sequencing is still a useful method

The SSM is nearly 40 years old, and it remains a useful molecular tool for genetic testing. It has its limitations because it is time-consuming, has limited use for long DNA fragments and is unable to detect sequences out of the region contemplated. We consider using “directed” SSM as first-line approach for diagnosis of suspected cases of ATS in places where NGS is not an option for genetic testing (due to low availability or high cost).
