# Supplementary material for: Sanger sequencing as a first-line approach for molecular diagnosis of Andersen-Tawil syndrome
Source: F1000Res. 2017 Jun 28;6:1016. [Version 1] doi: 10.12688/f1000research.11610.1 (PMC5635448; doi:10.12688/f1000research.11610.1)
Supplement: Peer review history

# Peer review: Referee response for version 1

**DOI:** 10.5256/f1000research.12541.r26270

**Published:** 2017-10-16

**Authors:** Diomedes E. Logothetis, Coeli M. Lopes

**Affiliations:** 1 Cardiovascular Research Institute, Department of Medicine, University of Rochester, Rochester, NY, USA; 2 Department of Pharmaceutical Sciences, School of Pharmacy, Bouve College of Health Sciences, Northeastern University, Boston, MA, USA

The opinion article by Totomoch-Serra and colleagues suggests that a “targeted” Sanger Sequencing Method (SSM) is an appropriate first-line approach to diagnose Andersen-Tawil syndrome (ATS). Although Next-Generation Sequencing (NGS) technology, especially in cases of genetic heterogeneity, is gradually replacing the traditional SSM as the method of choice for genetic testing, its high cost and unavailability often preclude its routine use, particularly in developing countries. The authors maintain that for conditions that require sequencing of short fragments of DNA (<1000 bases), previously amplified by PCR, SSM remains the gold standard. In ATS, where 60% of the reported cases involve mutations of the *KCNJ2* gene (that codes for the Kir2.1 channel protein) exhibiting autosomal dominant Mendelian inheritance, half the mutations are located in the C-terminal cytosolic domain with a hotspot at Arg218 (ATS type 1). Characteristic developmental abnormalities coupled to specific ventricular arrhythmias and/or muscular disorders can identify ATS patients who could then undergo SSM to identify the putative causative mutations in KCNJ2 with a potential 60% success. NGS could then be applied if the patient falls within the 40% of the cases that are not due to KCNJ2 (ATS type 2) with the hope to discover additional genes responsible for the disease and determine their frequency of occurrence. The opinion expressed by the authors is reasonable and advisable to physicians especially in cases where cost and access to NGS are problematic. Yet, it is likely a matter of time before cost and access to NGS improve and limit the choice of SSM for genetic testing. SSM has certainly served us well for the past 40 years and continues to serve us well in cases like ATS type 1 but it is inevitable that NGS will eventually make it obsolete. Until then, the authors make their case convincingly through this opinion article.

We have read this submission. We believe that we have an appropriate level of expertise to confirm that it is of an acceptable scientific standard.

# Peer review: Referee response for version 1

**DOI:** 10.5256/f1000research.12541.r26267

**Published:** 2017-10-06

**Authors:** Estelle Gandjbakhch

**Affiliations:** 1 Unité de Rythmologie, Institut de Cardiologie, Pitie Salpêtrière University Hospital, Paris, France

This opinion article by Totomoch-Serra *et al*.refers to the usefulness of directed sequencing of *KCNJ2* by Sanger method for molecular diagnosis of Andersen-Tawil syndrome (ATS), a rare QT long syndrome associating ventricular arrhythmias, prominent U wave, dysmorphic facial or skeletal features and periodic paralysis.

 Sanger sequencing remains a useful method for directed sequencing of specific clinical syndromes with low genetic heterogeneity as ATS, especially in places where NGS is not accessible. In another hand, NGS is particularly useful and cost effective in inherited cardiomyopathies or channelopathies associated with high genetic heterogeneity (like dilated cardiomyopathy) or caused by mutations in larges genes (as *RYR2* or *TTN*) where Sanger sequencing remains a long and expensive process. NGS can also be useful at second level when directed molecular diagnosis with Sanger method is negative.

 In case where ATS is suspected, the approach described by the authors with directed sanger sequencing at first line followed by NGS in case of negative screening is a cost-effective approach for ATS molecular diagnosis.

 I would only suggest describing in more details the clinical features (including symptoms or family history) suggestive of ATS diagnosis that should direct to *KCNJ2* sequencing at first line. Addition of a typical ECG could also be useful for the reader. Comparison of costs between NGS and Sanger sequencing could also be interesting.

I have read this submission. I believe that I have an appropriate level of expertise to confirm that it is of an acceptable scientific standard.

# Peer review: Referee response for version 1

**DOI:** 10.5256/f1000research.12541.r24341

**Published:** 2017-07-19

**Authors:** Oscar Campuzano

**Affiliations:** 1 Department of Medical Sciences, School of Medicine, University of Girona, Girona, Spain

It is a well written manuscript focused on use of Sanger technology in genetic diagnosis. Currently, despite to NGS technology allows a cost-effective analysis of hundreds genes in a reduced time, Sanger sequencing remains as gold standard for validation of variants identified using NGS, segregation of variants in family members, and analysis of small genes, such as KCNJ2.

I have read this submission. I believe that I have an appropriate level of expertise to confirm that it is of an acceptable scientific standard.
